# Defining the Role of Fluid Shear Stress in the Expression of Early Signaling Markers for Calcific Aortic Valve Disease

**DOI:** 10.1371/journal.pone.0084433

**Published:** 2013-12-23

**Authors:** Ling Sun, Nalini M. Rajamannan, Philippe Sucosky

**Affiliations:** 1 Department of Aerospace and Mechanical Engineering, University of Notre Dame, Notre Dame, Indiana, United States of America; 2 Eck Institute for Global Health, University of Notre Dame, Notre Dame, Indiana, United States of America; 3 Molecular Biology and Biochemistry, Mayo Clinic School of Medicine, Rochester, Minnesota, United States of America; Brigham and Women's Hospital, Harvard Medical School, United States of America

## Abstract

Calcific aortic valve disease (CAVD) is an active process presumably triggered by interplays between cardiovascular risk factors, molecular signaling networks and hemodynamic cues. While earlier studies demonstrated that alterations in fluid shear stress (FSS) on the fibrosa could trigger inflammation, the mechanisms of CAVD pathogenesis secondary to side-specific FSS abnormalities are poorly understood. This knowledge could be critical to the elucidation of key CAVD risk factors such as congenital valve defects, aging and hypertension, which are known to generate FSS disturbances. The objective of this study was to characterize *ex vivo* the contribution of isolated and combined abnormalities in FSS magnitude and frequency to early valvular pathogenesis. The ventricularis and fibrosa of porcine aortic valve leaflets were exposed simultaneously to different combinations of sub-physiologic/physiologic/supra-physiologic levels of FSS magnitude and frequency for 24, 48 and 72 hours in a double cone-and-plate device. Endothelial activation and paracrine signaling were investigated by measuring cell-adhesion molecule (ICAM-1, VCAM-1) and cytokine (BMP-4, TGF-β1) expressions, respectively. Extracellular matrix (ECM) degradation was characterized by measuring the expression and activity of the proteases MMP-2, MMP-9, cathepsin L and cathepsin S. The effect of the FSS treatment yielding the most significant pathological response was examined over a 72-hour period to characterize the time-dependence of FSS mechano-transduction. While cytokine expression was stimulated under elevated FSS magnitude at normal frequency, ECM degradation was stimulated under both elevated FSS magnitude at normal frequency and physiologic FSS magnitude at abnormal frequency. In contrast, combined FSS magnitude and frequency abnormalities essentially maintained valvular homeostasis. The pathological response under supra-physiologic FSS magnitude peaked at 48 hours but was then maintained until the 72-hour time point. This study confirms the sensitivity of valve leaflets to both FSS magnitude and frequency and suggests the ability of supra-physiologic FSS levels or abnormal FSS frequencies to initiate CAVD mechanisms.

## Introduction

As the underlying cause of 30,000 deaths annually and 55,000 hospital discharges, aortic valve disorders have tremendous economic and societal impact [1]. Calcific aortic valve disease (CAVD) remains the most common valvular condition and affects 26% of the population above 65 years of age [2]. The formation of calcific lesions on the valve leaflets contributes to the progressive obstruction of the left ventricular outflow and heart failure. The fundamental mechanisms that have been identified in the development of the disease include inflammation [3], [4], valvular interstitial cell activation [5], [6], remodeling [7]–[9], osteogenesis [10]–[12], apoptosis [13], [14] and necrosis [15], [16]. Valvular inflammation, which is the hallmark of the early stage of CAVD, has been linked to the activation of the leaflet endothelium via enhanced expression of cell adhesion molecules (VCAM-1, ICAM-1) [17]. Elevated levels of pro-inflammatory cytokines such as bone morphogenic proteins (BMPs) [18] and transforming growth factor-β1 (TGF-β1) [13], [14] have also been observed in early calcific lesions, demonstrating the key role played by paracrine signaling in CAVD development. Downstream of those events, the valve interstitial cells switch from a quiescent fibroblastic phenotype to an activated myofibroblastic or osteoblastic phenotype [8], [10], [19], [20]. Those activated phenotypes result in the progressive loss of valvular homeostasis caused by an overexpression of matrix metalloproteinases (MMPs) [7], [8] and cathepsins [21], [22], an increase in their enzymatic activity and the downregulation of their inhibitors (TIMPs) [23]. The ultimate development of calcific lesions is associated with elevated alkaline phosphatase activity and the upregulation of bone matrix proteins [24], [25].

While CAVD has been described historically as a passive degenerative process, it has now emerged as a highly regulated pathology presumably triggered by a combination of conventional cardiovascular risk factors [26], mechanical [16], [27], [28] and hemodynamic cues [29]–[31]. Fluid shear stress (FSS) is the frictional force acting in the direction of blood flow on the leaflet endothelium. FSS is experienced by the ventricularis when blood flows past the leaflets during systole and on the fibrosa when blood pools into the sinuses during diastole. Those different flow environments subject the leaflets to a side-specific FSS, which is unidirectional and pulsatile on the ventricularis and bidirectional and oscillatory on the fibrosa [32]–[34]. Recent evidence points to the existence of intimate interplays between the FSS environment and valvular biology. Physiologic FSS contributes to valvular homeostasis by regulating valvular biosynthetic activity [35], [36] and endothelial cell alignment [37], [38]. In contrast, FSS abnormalities have been shown to promote endothelial activation and leaflet inflammation [39], [40], two precursor events to CAVD. Despite the emerging evidence of the role played by FSS in valvulopathy, the mode of FSS transduction into a pathological response remains poorly understood. Specifically, it is not clear which FSS characteristic (i.e., magnitude, frequency) is preferentially sensed by the leaflets and what amount of disturbance is needed to trigger pathological events. This knowledge could be critical to the elucidation of key CAVD risk factors such as congenital valve defects, aging, and hypertension [2], [41], [42], which are associated with hemodynamic alterations that may stimulate the development of calcific lesions via disturbances in FSS magnitude and/or frequency. In fact, the bicuspid aortic valve, which is the most common congenital heart anomaly, generates significant FSS magnitude and frequency abnormalities due to the presence of two functional leaflets instead of three [32] and stimulates the development of calcific lesions at a more rapid rate than in the tricuspid aortic valve [43], [44]. Aging and hypertension, which also promote CAVD pathogenesis, result in progressive alterations in leaflet matrix stiffness [45] and transvalvular flow rate [46], respectively, that may subject the leaflets to increased FSS magnitude [47].

Therefore, the goal of this study was to determine for the first time the role of FSS magnitude and/or frequency abnormalities in early CAVD pathogenesis. The methodology involved the *ex vivo* exposure of porcine aortic valve leaflets to side-specific alterations in FSS magnitude and/or frequency and the biological assessment of the acute pathological response in terms of inflammation and remodeling via immunostaining, immunoblotting and zymography. The results reveal the key role played by FSS abnormalities in the active regulation of early CAVD pathogenic events.

## Materials and Methods

### Mechanical treatments and tissue conditioning

**Figure 1:**
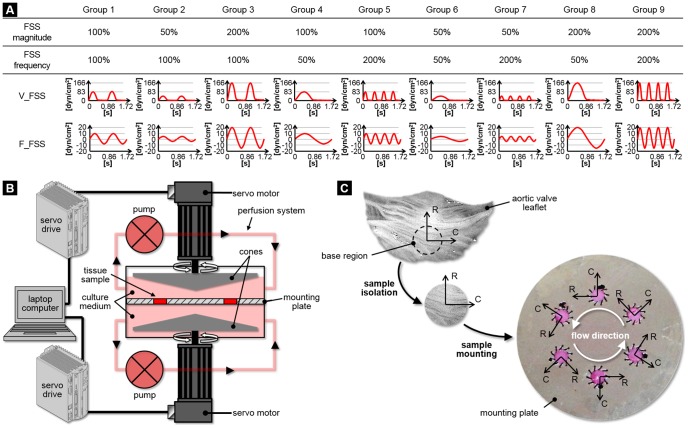
Experimental methods. Side-specific FSS conditions considered in this study (A), schematic of the shear stress device used to condition porcine aortic valve leaflet tissue to side-specific FSS (B) and tissue isolation and mounting (C) (V_FSS: ventricularis fluid shear stress, F_FSS: fibrosa fluid shear stress, R: radial direction, C: circumferential direction).

The investigation of the effects of FSS abnormalities on valvular pathobiology required the productions of the physiologic as well as abnormal FSS environments. As demonstrated *in vitro* [33], [34] and computationally [32], the native valvular FSS environment is side-specific and can be idealized as a unidirectional pulsatile shear stress varying between 0 and 80 dyn/cm^2^ on the ventricularis and a bidirectional oscillatory shear stress varying between −8 and +10 dyn/cm^2^ on the fibrosa. In addition to the physiologic FSS signal (group 1), eight abnormal FSS conditions were generated by decreasing/increasing the magnitude and/or frequency of the physiologic signal (groups 2–9; Fig. 1A). This analysis resulted in nine pairs of FSS waveforms (isolated effects of FSS magnitude: groups 1, 2, 3; isolated effects of FSS frequency: groups 1, 4, 5; combined effects of FSS magnitude and frequency: conditions 1, 6, 7, 8, 9), each pair corresponding to the temporal FSS variations experienced by the fibrosa and ventricularis. Experiments were conducted on fresh porcine leaflets using our double shear stress bioreactor [48] (Fig. 1B), which is capable of exposing simultaneously but independently both leaflet surfaces to side-specific FSS. Fresh porcine valves (6–12 months) were obtained from a local abattoir (Martin's Custom Butchering, Wakarusa, IN; permission was obtained from this slaughterhouse to use these animal parts), immediately transported to the laboratory in ice-cold phosphate buffered saline. A circular section of 7 mm in diameter was excised from the base region (i.e., region most prone to calcification [4]) of each leaflet (Fig. 1C). Two samples from each valve were used as experimental samples while the third sample served as fresh control. Six experimental samples were sutured to the plate of the bioreactor, exposing both their aortic and ventricular sides to flow. The native alignment of the tissue relative to blood flow was maintained by aligning the radial direction of the samples with the direction of the cone motion (Fig. 1C). The bioreactor was filled with culture medium (Dulbecco's Modified Eagle's Medium) supplemented with 3.7 g/L sodium bicarbonate, 0.05 g/L ascorbic acid, 10% non-essential amino acid solution, 5 mg/L bromodeoxyuridine and 1% penicillin-streptomycin. The established acute sensitivity of valvular tissue to mechanical stimulation [49] along with our objective to characterize the early pathological response provided the rationale for conditioning the leaflets for 48 hours. Following the biological assessment of all tissue groups, the mechanical treatment producing the most significant response was identified and two additional experiments were performed over 24 and 72 hours to characterize the time-dependence of the FSS-mediated biological response.

### Immunostaining

Frozen tissue sections were flash frozen in optimum cutting temperature media. The slides were first thawed to room temperature and then blocked using 10% animal serum in PBS (Sigma-Aldrich), 0.2% Trixon-100 (Sigma-Aldrich) and 1% dimethyl sulfoxide (DMSO; Thermo Fisher Scientific) in 1x PBS for 1 hour at room temperature. Following the blocking step, the slides were then incubated overnight at 4°C in primary antibody in 2–10% blocker at the following dilutions: VCAM-1 (1∶50; Santa Cruz Biotechnology), ICAM-1 (1∶50; Southern Biotech), TGF-β1 (1∶25; Santa Cruz), BMP-4 (1∶150; Abcam), cathepsin L (1∶25; Santa Cruz Biotechnology), cathepsin S (1∶25; Santa Cruz Biotechnology), MMP-2 (1∶100; EMD Millipore) and MMP-9 (1∶100; EMD Millipore). Following primary antibody incubation, sections were washed 3 times in 1x PBS and incubated with anti-rabbit or anti-mouse (all from Santa Cruz) secondary antibodies at 1∶100 dilution for 2 hours at room temperature. The tissue sections were then washed 3 times in 1x PBS, counterstained with 1 4′,6-Diamidino-2-phenylindole (DAPI; Sigma-Aldrich), mounted with fluorescence mounting medium (Dako), coverslipped and stored at 4°C. Slides were subsequently imaged under the mercury lamp of a Nikon E600 imaging microscope using a TR/FITC/DAPI filter.

### Immunoblotting

Following each mechanical treatment, the specimens were pulverized using a mortar and pestle in liquid nitrogen, homogenized in ice-cold RIPA buffer (Santa-Cruz) and centrifuged at 7,000 g to pellet extracellular matrix debris for 8 minutes at 4°C. The supernatant was assayed for protein concentration using a bicinchoninic acid (BCA) protein assay (Pierce). Equal amounts of tissue lysates were resolved by reducing SDS-PAGE. After transfer to a polyvinylidene difluoride (PVDF) membrane (EMD Millipore) using a mini trans-blot cell (Bio-Rad), the blots were blocked with 5% non-fat drymilk and probed with a primary antibody against BMP-4 (1∶500, Abcam), TGF-β1 (1∶200, Santa Cruz). Depending on the primary antibody, appropriate anti-rabbit and anti-mouse HRP secondary antibody (1∶2000, Santa Cruz) was then used. The membranes were finally incubated in horseradish peroxidase-conjugated streptavidin. Immunopositive bands were then detected using a luminol-based chemiluminescence reagent (Pierce) against standard radiography film in a darkroom. The films were then analyzed by densitometry using ImageJ (NIH).

### Gelatin zymography

Zymography was performed to quantify the activity of the proteolytic enzymes MMP-2 and MMP-9. Equal amounts of tissue lysates assayed by BCA were resolved by sample buffer (Bio-Rad) and loaded in the gel (Bio-Rad). After running, the gels were washed in renaturation buffer (Bio-Rad) and developing buffer (Bio-Rad). The gels were stained by adding stain solution (Sigma-Aldrich), de-stained in deionized water and scanned. The images were then analyzed by densitometry using ImageJ.

### Semi-quantification of immunostained images

The semi-quantitative assessment of immunostained images was carried out using ImageJ. Briefly, the intensities of positively stained regions were estimated and normalized by the number of cells visible in the images to yield a quantity consistent to a biomarker expression per cell. Distinction was not made between endothelial and interstitial cells during the cell counting.

### Densitometric assessment of immunoblots and zymograms

The dedicated macro of ImageJ was used to plot the histogram of individual lanes/bands in blots/gels. These histograms were then integrated to obtain the mean intensity of each immune-positive band. For the blots, these intensities for a particular protein were then normalized by the intensity values for β-actin (Santa Cruz), which was used as a housekeeping protein.

### Statistical analyses

Each experimental group consisted of six leaflet samples. All quantitative data were expressed as means ± SE and then normalized with respect to the values measured in fresh tissue (control). Data from all experiments were first tested for normality by the Anderson-Darling method, then analyzed using ANOVA to determine if there was significant contribution by a particular mechanical treatment on the measured parameters, followed by the Bonferroni post-hoc test. A p-value of less than 0.05 was used as a measure of statistical significance. All statistical analyses were performed using SPSS (IBM).

### Pro-inflammatory and remodeling state indices

The net effect of a particular FSS environment on pro-inflammatory processes after 48 hours of conditioning was estimated by calculating a pro-inflammatory state index 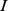 defined as the product of the fold change in expression 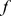 of each pro-inflammatory mediator (ICAM-1, VCAM-1, BMP-4, TGF-β1) with respect to fresh tissue: 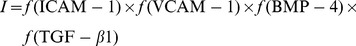
(1)


Similarly, the contributions of FSS abnormalities to valvular remodeling were quantified via a tissue remodeling index 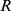 defined as the product of the fold change in expression 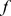 of each ECM remodeling marker (MMP-2, MMP-9, cathepsin-S and cathepsin-L) relative to fresh tissue:
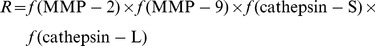
(2)


An index smaller than one indicates a net decrease in pro-inflammatory/remodeling response, while an index larger than one reflected a net increase in tissue pro-inflammatory/remodeling processes. The nine pro-inflammatory and remodeling state indices resulting from the nine FSS magnitude-frequency treatments were interpolated using a biharmonic spline in order to predict the dependence of the tissue pathologic state on FSS magnitude and frequency.

## Results

### Side-specific FSS abnormalities do not promote endothelial activation

**Figure 2:**
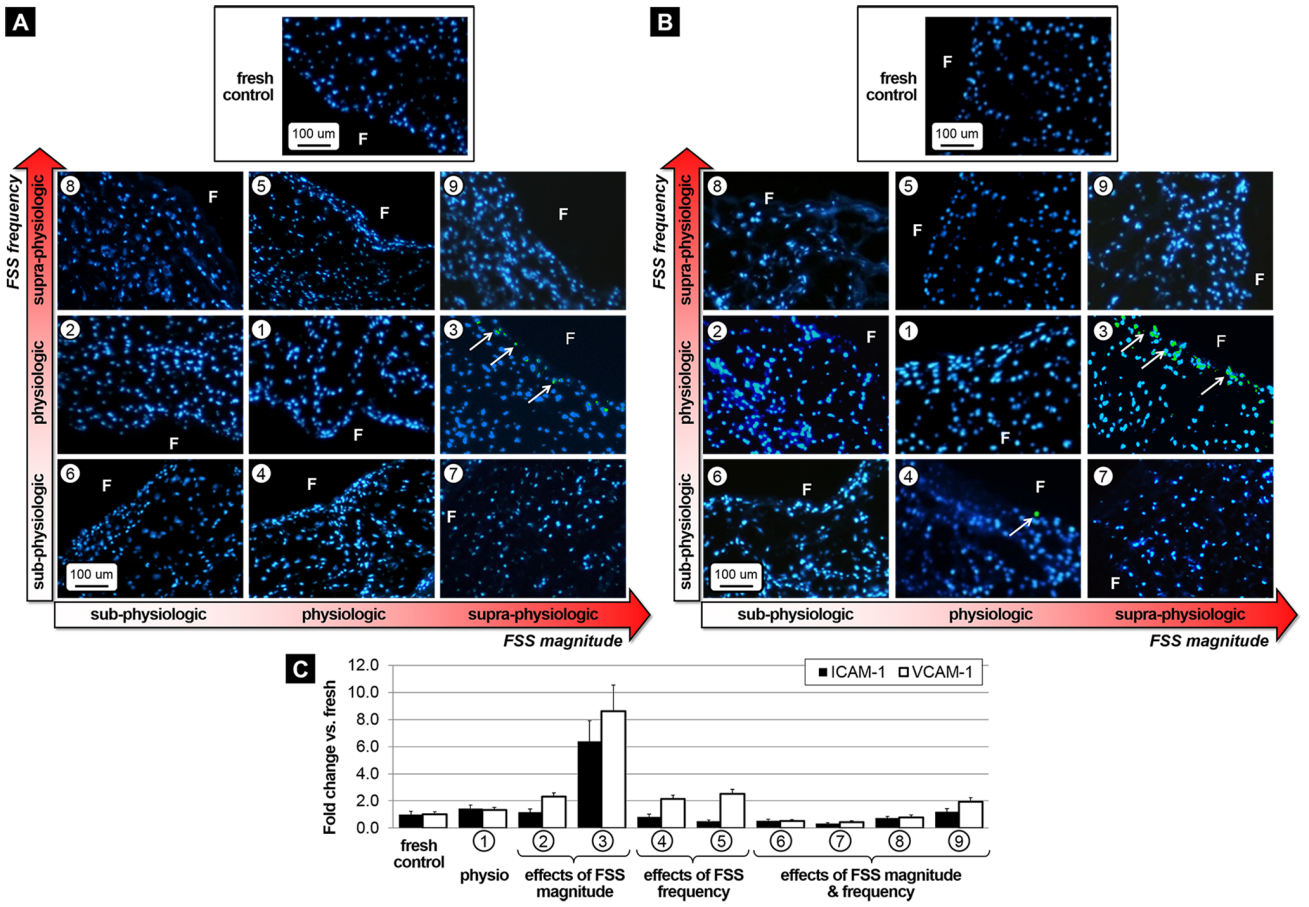
Effects of FSS abnormalities on endothelial activation. Immunostaining for ICAM-1 (A) and VCAM-1 (B) after FSS conditioning for 48 hours and semi-quantitative results (C) (F: fibrosa, blue: cell nuclei; green: positively stained cells).

Following tissue exposure to physiologic and non-physiologic levels of FSS magnitude and frequency, immunostaining was performed to examine endothelial activation. Positive staining for ICAM-1 (Fig. 2A) and VCAM-1 (Fig. 2B) was only detected on the endothelial lining of the fibrosa in tissue subjected to supra-physiologic FSS magnitude and physiologic frequency (group 3). Although the results are supported by the semi-quantitative analysis that suggests a 6.4-fold and 8.6-fold increase in ICAM-1 and VCAM-1 expression, respectively, relative to the fresh controls, and a 4.5-fold and 6.5-fold, respectively, relative to the physiologic FSS treatment (Fig. 2C), those changes were not statistically significant (p>0.05). All quantitative results are summarized in Table 1.

**Table 1:** Fold change in expression of each biomarker relative to fresh control for all mechanical treatments after 48 hours of conditioning and quantitative assessment method (WB: western blot; IHC: immunohistochemistry; * p<0.05 vs. fresh control, # p<0.05 vs. physiologic FSS).

		Tissue groups									
Biomarker	Method	1	2	3	4	5	6	7	8	9	
ICAM-1	IHC	142%	116%	640%	82%	49%	53%	32%	73%	119%	
VCAM-1	IHC	132%	231%	861%	214%	252%	53%	42%	78%	195%	
BMP-4	WB	166%	106%	498%*	144%	116%	141%	126%	18%*^#^	21%*^#^	
TGF-β1	WB	40%	192%	474%*^#^	102%	111%	58%	87%	20%*^#^	17%*^#^	
MMP-2	IHC	106%	450%	762%*^#^	1048%*^#^	1000%*^#^	136%	172%	31%	30%	
MMP-9	IHC	62%	272%	939%*^#^	968%*^#^	997%*^#^	103%	58%	35%	32%	
cathepsin-L	IHC	130%	95%	1111%*^#^	469%	480%	161%	572%	327%	290%	
cathepsin-S	IHC	86%	105%	449%*^#^	164%	391%*^#^	71%	8%	14%	37%	

### Supra-physiologic FSS magnitude promote paracrine signaling via BMP-4- and TGF-β1-dependent pathways

**Figure 3:**
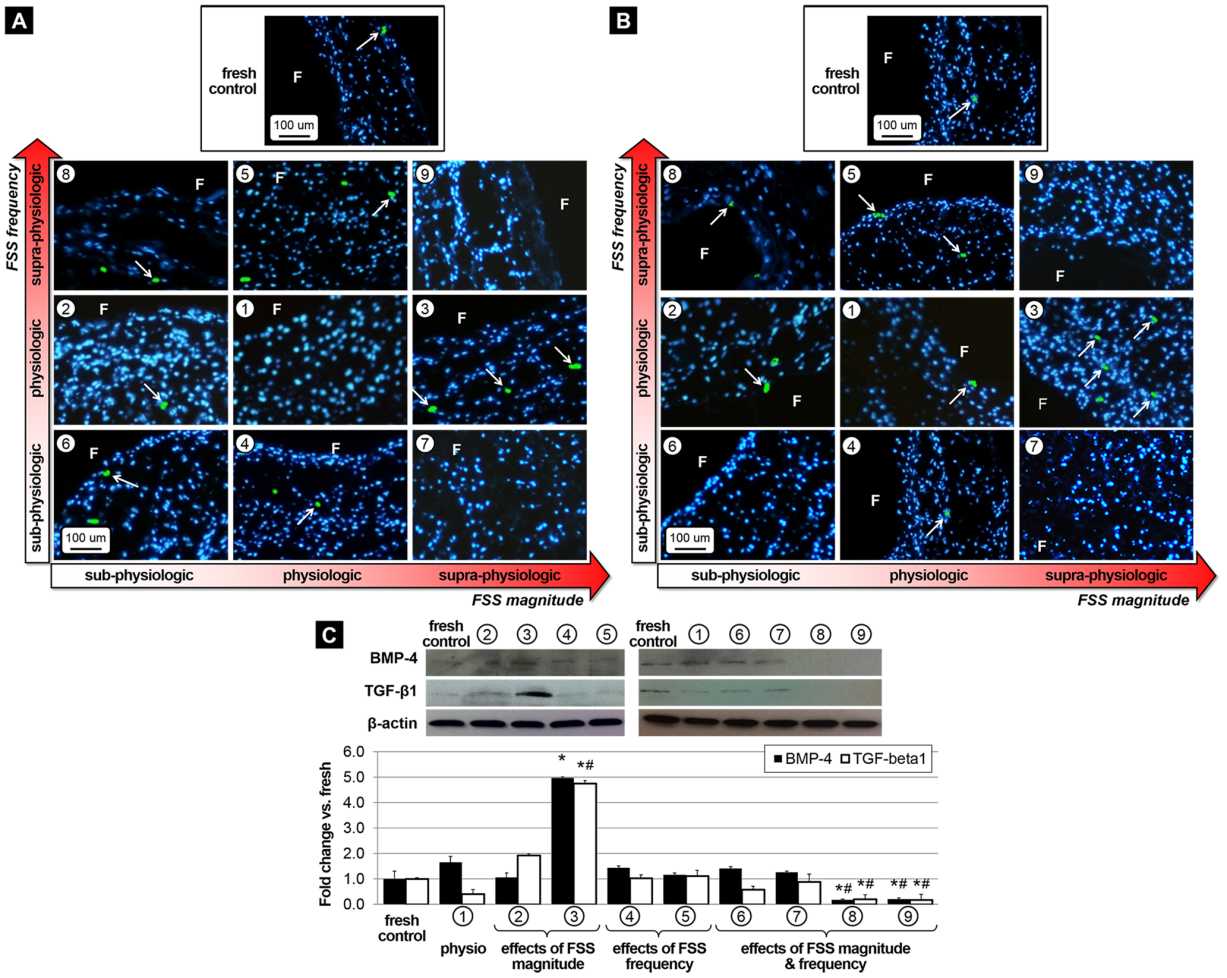
Effects of FSS abnormalities on paracrine signaling. Immunostaining for BMP-4 (A) and TGF-β1 (B) after FSS conditioning for 48 hours, and Western blot results with densitometric assessment (C) (F: fibrosa, blue: cell nuclei, green: positively stained cells, * p<0.05 vs. fresh control, # p<0.05 vs. physiologic FSS).

BMP-4 and TGF-β1 immunostaining and immunoblotting were performed to investigate the effects of FSS abnormalities on paracrine signaling. Similar to the cell-adhesion molecules, BMP-4 and TGF-β1 expression (Fig. 3A and 3B, respectively) was detected in the sub-endothelial layer of tissue exposed to supra-physiologic FSS magnitude at normal frequency (group 3). Those observations are supported by the Western blot results (Fig. 3C) which suggest that supra-physiologic FSS magnitude (group 3) resulted in a significant 5-fold increase in BMP-4 and TGF-β1 expression relative to fresh controls. Tissue exposure to physiologic FSS (group 1) essentially maintained the BMP-4 and TGF-β1 expression levels measured in fresh controls. In addition, tissue exposure to abnormal frequencies of elevated FSS (groups 8 and 9) significantly decreased the expression of the cytokines with respect to the fresh controls (group 8: 82% and 80% decrease in BMP-4 and TGF-β1 expression, respectively; group 9: 79% and 83% decrease in BMP-4 and TGF-β1 expression, respectively) and the tissue conditioned under physiologic FSS (group 8: 89% and 50% decrease in BMP-4 and TGF-β1 expression, respectively; group 9: 87% and 58% decrease in BMP-4 and TGF-β1 expression, respectively). All quantitative results are summarized in Table 1.

### Abnormalities in FSS magnitude or frequency promote ECM remodeling via MMP- and cathepsin-dependent pathways

**Figure 4:**
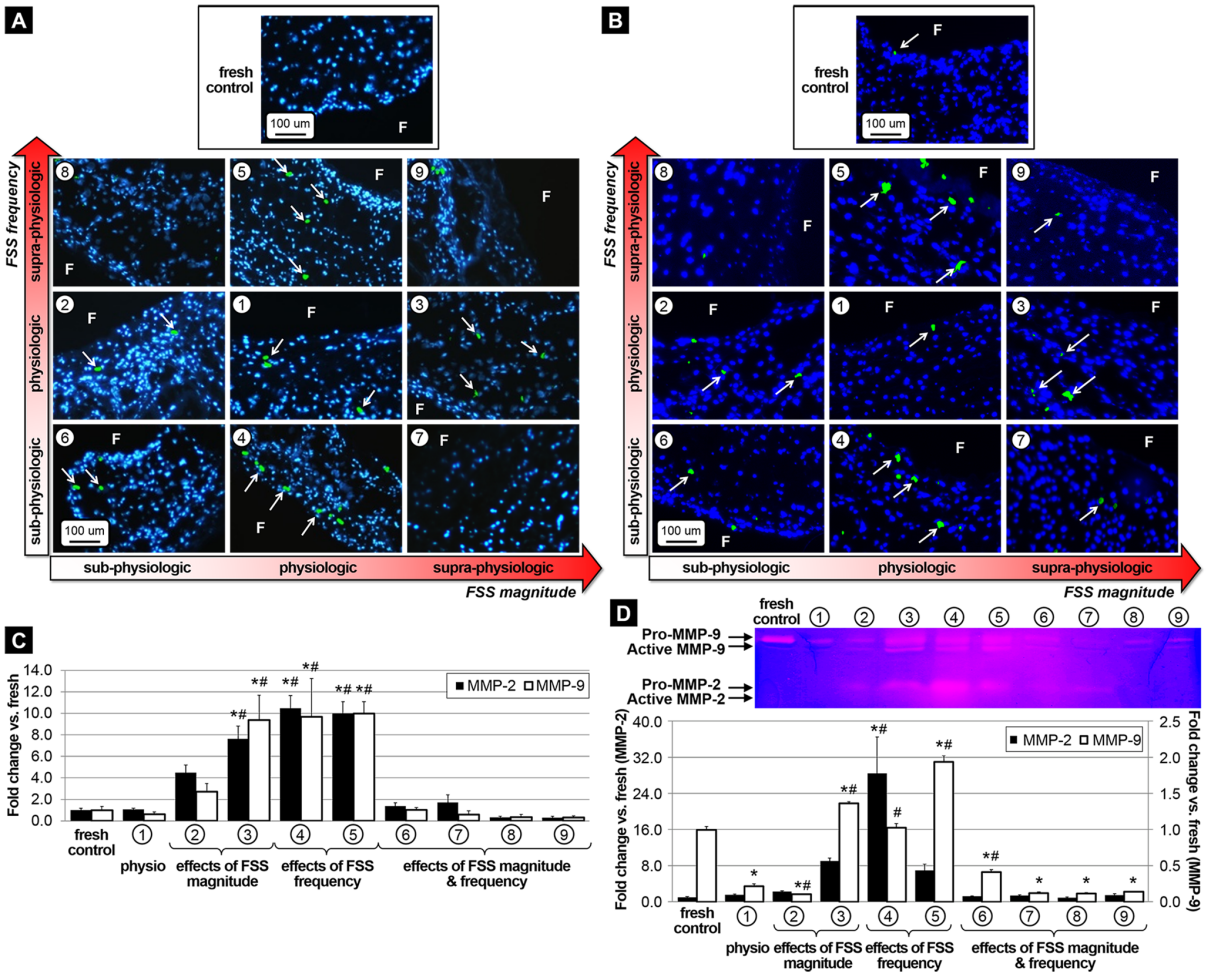
Effects of FSS abnormalities on proteolytic enzyme expression and activity. Immunostaining for MMP-2 (A) and MMP-9 (B) after FSS conditioning for 48 hours, semi-quantitative results (C) and zymography results with densitometric assessment (D) (F: fibrosa, blue: cell nuclei; green: positively stained cells, * p<0.05 vs. fresh control, # p<0.05 vs. physiologic FSS).

**Figure 5:**
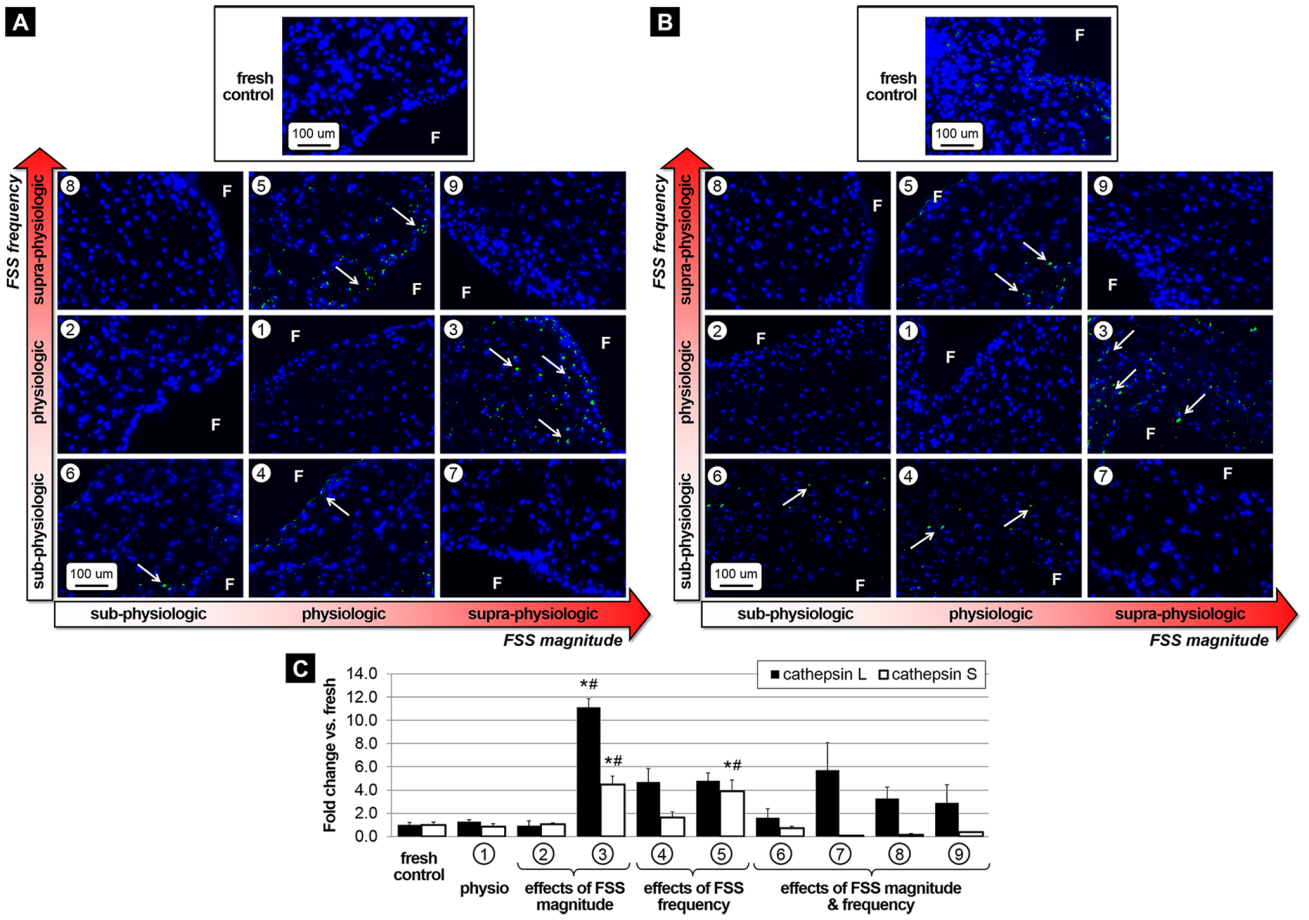
Effects of FSS abnormalities on elastolytic protease expression. Immunostaining for cathepsin-L (A) and cathepsin-S (B) after FSS conditioning for 48 hours and semi-quantitative results (C) (F: fibrosa, blue: cell nuclei; green: positively stained cells, * p<0.05 vs. fresh control, # p<0.05 vs. physiologic FSS).

The effects of FSS abnormalities on valvular remodeling were investigated by comparing the expression of the proteolytic enzymes MMP-2 and MMP-9 and the elastolytic proteases cathepsin L and cathepsin S. MMP-2 and MMP-9 immunostaining (Fig. 4A and 4B, respectively) suggest the maintenance of normal enzymatic expression in response to physiologic FSS (group 1) and combined alterations in FSS magnitude and frequency (groups 6–9). In contrast, elevated FSS magnitude (group 3) and abnormalities in FSS frequency (groups 4 and 5) resulted in increased proteolytic enzyme expression relative to fresh controls. The semi-quantitative results (Fig. 4C) support those observations. Tissue from groups 3, 4 and 5 exhibited a significant increase in MMP-2 expression relative to both fresh tissue (7.6-fold, 10.5-fold and 10.0-fold, respectively) and tissue subjected to physiologic FSS (7.2-fold, 9.9-fold and 9.4-fold, respectively). MMP-9 expression was significantly increased in response to supra-physiologic FSS magnitude at normal frequency (group 3) and isolated abnormalities in FSS frequency (groups 4 and 5) relative to fresh tissue (9.4-fold, 9.7-fold and 10.0-fold, respectively) and tissue conditioned under physiologic FSS (15.1-fold, 15.6-fold and 16.1-fold, respectively). Gelatin zymography was carried out to quantify MMP-2 and MMP-9 activity in the different tissue groups (Fig. 4D). The densitometric analysis of the zymograms revealed that physiologic FSS (group 1) maintained MMP-2 activity but elicited a significant 78% decrease in MMP-9 activity with respect to fresh control. Tissue from group 4 exhibited a 28.6-fold increase in MMP-2 activity as compared to fresh tissue (p<0.05) and a 18.5-fold increase relative to tissue exposed to physiologic FSS (p<0.05). There was also a significant increase in MMP-9 activity in tissue from groups 3 and 5 as compared to fresh tissue (1.4-fold and 2.0-fold, respectively) and tissue conditioned under physiologic FSS (6.2-fold and 8.9-fold, respectively). In contrast, combined alterations in FSS magnitude and frequency (groups 6–9) were associated with the maintenance of MMP-2 activity but a significant decrease in MMP-9 activity (0.4-fold, 0.1-fold, 0.1-fold and 0.1-fold change, respectively) relative to fresh tissue. While cathepsin L and cathepsin S expression was not detected in response to physiologic FSS (Fig. 5A and 5B, respectively), widespread positive staining was observed under supra-physiologic FSS magnitude at normal frequency (group 3) and supra-physiologic FSS frequency at normal magnitude (group 5). The semi-quantitative results (Fig. 5C) indicate a significant 11.1-fold and 8.5-fold increase in cathepsin L expression in tissue exposed to supra-physiologic FSS (group 3) relative to fresh controls and tissue subjected to physiologic FSS, respectively. Although cathepsin L was upregulated in response to combined abnormalities in FSS magnitude and frequency (groups 6–9), the response was not statistically different from that in fresh controls. Cathepsin S expression measured in tissue from groups 3 and 5 was significantly increased relative to fresh controls (4.5-fold and 3.9-fold, respectively) and tissue exposed to physiologic FSS (5.2-fold and 4.5-fold, respectively). All quantitative results are summarized in Table 1.

### Pro-inflammatory and remodeling indices

**Figure 6:**
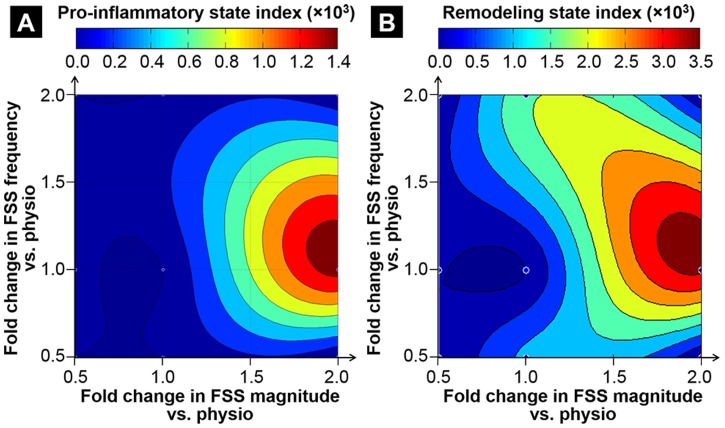
Correlation between FSS abnormalities and leaflet pathological state. Pro-inflammatory state index (A) and remodeling state index (B) after 48 hours of FSS conditioning.

With most pro-inflammatory state index variations confined in the supra-physiologic FSS magnitude range, the results suggest the particular vulnerability of leaflet inflammation to elevated FSS levels (Fig. 6A). The symmetry of the pro-inflammatory state index distribution also suggests that, at a given FSS magnitude, pro-inflammatory pathways are equally sensitive to an increase or a decrease in FSS frequency. The FSS environment maximizing the pro-inflammatory state index consists of a supra-physiologic FSS magnitude (i.e., 200% physiologic level) at a relatively moderate FSS frequency (i.e., 112% physiologic level). Similarly, the analysis of the remodeling state index distribution reveals the strong dependence of valvular remodeling processes on elevated FSS magnitude (Fig. 6B). However, the skewness of the distribution toward the upper left quadrant suggests the higher sensitivity of those processes to supra-physiologic than to sub-physiologic FSS frequencies. The FSS environment maximizing the remodeling state index consists of a supra-physiologic FSS magnitude (i.e., 194% physiologic level) at a relatively moderate FSS frequency (i.e., 115% physiologic level).

### Supra-physiologic FSS magnitude increases vulnerability to CAVD in a time-dependent manner

**Figure 7:**
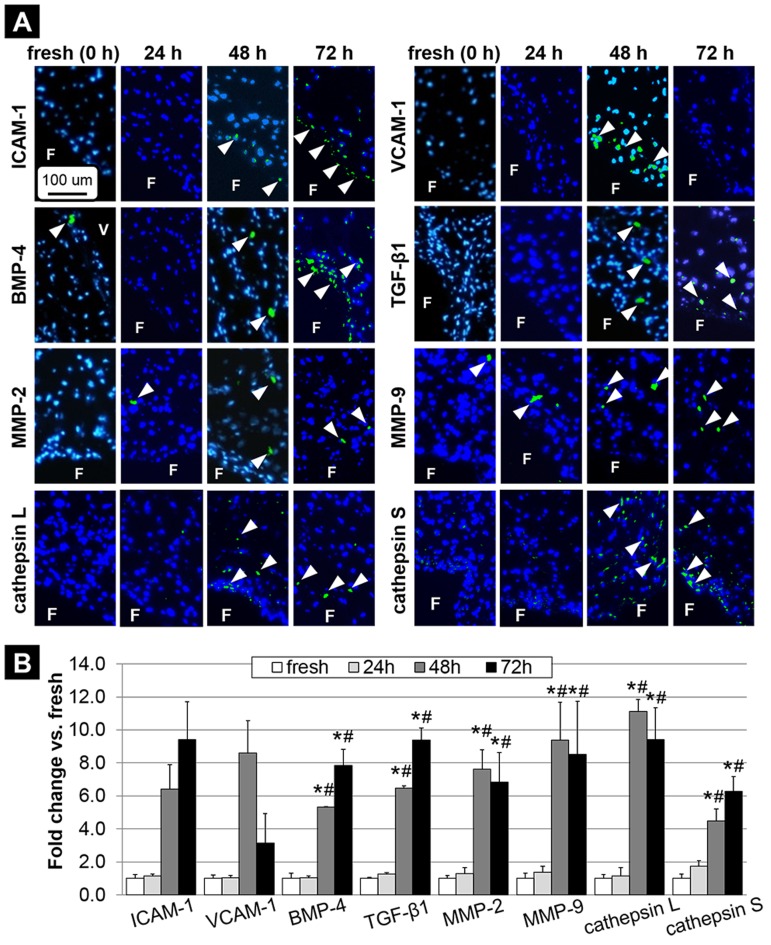
Time-dependence of pro-inflammatory and ECM remodeling biomarker expression following tissue exposure to supra-physiologic FSS magnitude under normal frequency for 24, 48 and 72 hours. Immunostaining (A) and semi-quantitative results (B) (F: fibrosa, V: ventricularis, blue: cell nuclei; green: positively stained cells, * p<0.05 vs. fresh control, # p<0.05 vs. 24 h).

The time-dependence of the leaflet pathological response following conditioning to supra-physiologic FSS under normal frequency (i.e., FSS environment shown to be most conducive to early CAVD mechanisms) was studied by assessing the biological response of tissue exposed to this environment for 24 and 72 hours and by comparing it to those obtained in the fresh controls and tissue conditioned for 48 hours (Fig. 7A). The semi-quantitative of the stained images (Fig. 7B) revealed no significant difference in the expression of all biomarkers between the fresh controls (i.e., 0-hour time point) and the 24-hour time point. In contrast, while ICAM-1 and VCAM-1 expressions did not increase significantly between 24 and 48 hours of FSS conditioning, all other biomarkers were significantly upregulated between those two time points (5.2-fold, 5.1-fold, 5.9-fold, 6.9-fold, 9.6-fold and 2.6-fold increase in BMP-4, TGF-β1, MMP-2, MMP-9, cathepsin L and cathepsin S, respectively). Interestingly, the expression of all biomarkers was essentially maintained beyond the 48-hour time point as no further significant change occurred between 48 and 72 hours of conditioning. All quantitative results are summarized in Table 2.

**Table 2:** Fold change in expression of each biomarker relative to fresh control after 24, 48 and 72 hours of conditioning under supra-physiologic FSS magnitude (group 2) and quantitative assessment method (WB: western blot; IHC: immunohistochemistry; * p<0.05 vs. fresh control, # p<0.05 vs. 24 h).

		Time points		
Biomarker	Method	24 hours	48 hours	72 hours
ICAM-1	IHC	116%	640%	942%
VCAM-1	IHC	104%	861%	315%
BMP-4	IHC	103%	498%*^#^	784%*^#^
TGF-β1	IHC	127%	474%*^#^	738%*^#^
MMP-2	IHC	130%	762%*^#^	682%*^#^
MMP-9	IHC	137%	939%*^#^	851%*^#^
cathepsin-L	IHC	116%	1111%*^#^	941%*^#^
cathepsin-S	IHC	175%	449%*^#^	628%*^#^

## Discussion

The objective of this study was to elucidate the respective and combined effects of abnormalities in FSS magnitude and frequency on the early mechanisms of CAVD in porcine aortic valve leaflets. The key results of the study can be summarized as follows: 1) leaflet tissue is selectively more sensitive to alterations in FSS magnitude than frequency; 2) FSS abnormalities promote paracrine signaling via BMP-4 and TGF-β1-dependent pathways and ECM degradation via MMP- and cathepsin-dependent pathways; and 3) the FSS mechanisms of early CAVD development are time-dependent. Therefore, the results confirm the key contribution of FSS alterations to valvular pathogenesis and suggest the capability of FSS abnormalities to trigger early CAVD events in the absence of any other risk factor.

The focus of this study on the characterization of the acute effects of FSS abnormalities on endothelial activation, paracrine signaling and ECM degradation is motivated by the established involvement of those biological processes in CAVD pathogenesis [4], [50] and the possible pathophysiological relevance of FSS abnormalities to disease onset and early progression. Many CAVD risk factors are associated directly or indirectly to progressive alterations in the valvular hemodynamic environment and the FSS experienced by the leaflets. Aging, for example, is accompanied by the progressive stiffening of the leaflet ECM, which has been shown to produce elevated FSS magnitude on the leaflets [47]. The congenital bicuspid aortic valve exposes certain regions of its leaflets to a high FSS magnitude and frequency environment [32], which has been shown to promote early CAVD pathogenesis [51]. Therefore, the particular vulnerability of valvular inflammation and remodeling to FSS magnitude and frequency abnormalities evidenced in this study may explain, at least partially, the susceptibility of an aging or anatomically abnormal valve to calcify. It is important to note that an abnormal FSS frequency per se is not an alteration likely to occur *in vivo*. Tachycardia conditions, which may result in acute FSS frequency alterations, are not chronic and therefore unlikely to contribute to a long-term pathological response. However, the rationale for varying the frequency of the FSS signal was to investigate the effects of changes in the harmonics of the FSS waveform experienced locally by the leaflets. Such phenomenon has been evidenced on bicuspid aortic valve leaflets [32], [52] and is also expected to occur with hypertension and aging, i.e., two known CAVD risk factors that affect leaflet dynamics and are likely to generate local alterations in the harmonics of the FSS signal.

Experimental *in vivo* and *in vitro* models of CAVD proposed to date have relied on two leading hypotheses. The cholesterol hypothesis focuses on the traditional risk factors to study the biological events leading to CAVD [53]–[56]. In contrast, the hemodynamic hypothesis, which is foundation of the present study, assumes that mechanical stresses have the ability to activate these disease pathways. These divergent hypotheses have been reviewed in two theories on the development of CAVD: the LDL-density-radius theory [57] and the LDL-density-pressure theory [58], which account for the potential differences in timing and initiation events in the development of CAVD. While these theories have pointed out the important differences between the signaling of mechanical stresses and hypercholesterolemia, they have also suggested their intricate synergies. Therefore, the elucidation of CAVD pathogenesis requires the exploration of both the lipid and mechanical stress mechanisms of CAVD.

Our results demonstrated the particular sensitivity of valvular pro-inflammatory pathways to elevated FSS levels as illustrated by the increased expressions of the cytokines BMP-4 and TGF-β1. The ability of supra-physiologic mechanical stress environments to trigger pro-inflammatory processes has been observed *ex vivo* in the contexts of elevated pressure, stretch and FSS. Exposure of porcine aortic valve leaflets to hypertensive levels in a pressure chamber was shown to upregulate pro-inflammatory gene networks in interstitial cells [59]. In another study, the long-term (14 days) *ex vivo* exposure of porcine valve leaflets to elevated stretch with an osteogenic culture medium demonstrated stretch magnitude-dependent tissue mineralization mediated by BMP-4 and TGF-β1 pathways [60]. Similarly to elevated stretch and pressure, the present results indicate that supra-physiologic FSS magnitudes increase the vulnerability of valvular tissue to calcification via BMP-4- and TGF-β1-dependent pathways. Therefore, our findings suggest the pathogenic nature of elevated FSS magnitudes and their potential role as a trigger of mechanosensitive events leading to CAVD initiation and development. Those results are also consistent with previous *in vivo* evidence of the role played by BMP-4 in CAVD pathogenesis [61]–[63].

Our study also indicated increases in elastolytic enzyme expression and proteolytic enzyme expression and activity under elevated FSS magnitude (at normal frequency) and abnormal FSS frequency (at normal magnitude), suggesting the progressive loss of valvular homeostasis and the alteration of valvular biosynthetic activity. The ability of supra-physiologic mechanical stress environments to break the delicate balance between ECM synthesis and degradation has been observed previously in the contexts of elevated pressure, stretch or a combination of these. Hypertensive levels have been shown *ex vivo* to promote collagen and sulfated glycosaminoglycan (sGAG) production but to decrease cathepsin L, MMP-2 and MMP-9 expression and activity in porcine leaflets [35], [64]. Elevated strain levels have been shown to reduce sGAG content but to promote cellular proliferation and apoptosis, collagen content and proteolytic enzyme expression and activity in porcine leaflets [65]. Lastly, valve leaflets subjected *ex vivo* to combined pathological levels of pressure and stretch exhibit increased ECM synthesis and a progressive switch of the valve interstitial cells from a contractile to a more synthetic phenotype [66]. Consistent with those observations, the present study suggests that the expression and biosynthetic activity of some proteolytic enzymes may be mechanosensitive and play a key role in the progression of CAVD under altered mechanical loading.

It is important to note that the immunofluorescence images included in the paper focused on the fibrosa side due to the complete absence of positive stain on the ventricularis. Depending on the mechanical treatment, protein and enzyme expressions were distributed either in the subendothelial layer of the fibrosa or deeper in the tissue. Those results may correlate with the previously established phenotypic difference between the endothelial cells lining the leaflet fibrosa and ventricularis [29].

An interesting result is the apparent synergistic effects of FSS magnitude and frequency abnormalities on ECM degradation and paracrine signaling. In fact, leaflets exposed to physiologic FSS or combined abnormalities in FSS magnitude and frequency (i.e., four corners of the maps shown in Fig. 6) exhibited low pro-inflammatory and remodeling state index values. Interestingly, while leaflet exposure to elevated FSS magnitude (at normal frequency) produced the most significant pathological response, the combination of elevated FSS magnitude and abnormal frequency returned the expressions of cell-adhesion molecules, pro-inflammatory cytokines and proteases to their physiologic baseline levels. The same attenuating effect between mechanical stress magnitude and frequency has been observed in valvular response to cyclic pressure, for which collagen and sGAG syntheses were increased in response to elevated pressure magnitude and frequency, but to a lesser extent than in response to elevated pressure at normal frequency [67]. Those results highlight the complexity of valvular mechanobiology and the sensitivity of valvular tissue to both the magnitude and frequency of mechanical stress signals.

The implementation of a double cone-and-plate bioreactor in this study enabled the simultaneous exposure of the leaflet fibrosa and ventricularis to their respective native FSS directionality. Therefore, our study is the first one to investigate the effects of side-specific FSS on valvular biology. However, the isolated effects of FSS magnitude on valvular cytokine expression and endothelial activation have been studied previously using a single-sided shear stress bioreactor subjecting the fibrosa to mild and severe supra-physiologic FSS levels [40]. The exposure of the fibrosa to increasing FSS levels while maintaining the ventricularis under a static flow condition resulted in the same FSS magnitude-dependent increase in cytokine expression as that observed in the present study. In contrast, the non-significant increase in VCAM-1 and ICAM-1 expressions suggested by our results in response to elevated FSS contrasts with the significant endothelial activation response observed in that study following the exposure of the fibrosa to mild and severe supra-physiologic FSS magnitudes. We speculate that the absence of significant endothelial dysfunction in the present study may be due to the implementation of a more physiologic shear stress bioreactor, which subjected simultaneously both leaflet surfaces to side-specific FSS.

Another contribution of the study is the elucidation of the timescale required for the transduction of valvular FSS into a biological response. While the results indicate that the FSS-mediated pathological response after 24 hours of conditioning was essentially similar to that obtained in fresh tissue (i.e., initial time point), cytokine and protease expressions peaked after 48 hours of exposure to FSS and then remained relatively constant until the 72-hour mark. Similar temporal responses have been observed previously in the contexts of valvular mechano-response to cyclic FSS [39], [40], [51] and stretch [65], [68]. Those results support the previous evidence that 48 hours of conditioning are necessary and sufficient for the initial transduction of mechanical abnormalities into a pathological response [5]. Interestingly, the temporal response also indicates that, while it was not significant, the activation of the leaflet endothelium also occurred at 48 hours and therefore did not precede the upregulation of cytokines and proteases. This result is consistent with our earlier *ex vivo* study on the single-sided effects of FSS magnitude on valvular biology, which indicated that the pharmacological supplementation or inhibition of TGF-β1 had a dramatic effect on adhesion molecule and BMP-4 expressions but BMP-4 supplementation and inhibition resulted in a milder effect on the overall pathological response [40]. Those results and the observations described in the present study support the upstream role of paracrine signaling in the FSS-mediated response relative to endothelial activation mechanisms.

The comparison of the results obtained in the fresh control group and in the group exposed to physiologic FSS for 48 hours (group 1) revealed no statistical difference in the expression of the different biomarkers considered in the study. Only MMP-9 activity was significantly downregulated under physiologic FSS as compared to the control group (78% decrease; see Fig. 4D). To understand the significance of this result, it is important to note that the valve functions in a complex mechanical environment consisting of cyclic stretch, pulsatile pressure and FSS [49]. Therefore, our study suggests that, in the absence of any other mechanical stimulus, FSS might be able to maintain relatively normal valvular homeostasis over 48 hours. This result is consistent with the previous demonstration of the constant renewal of valvular tissue in response to mechanical stimulation [69] and indicates that, while the full spectrum of mechanical signals may be needed to maintain valvular homeostasis, FSS may play a dominant role over other forces in this process.

Finally, it is important to note that our experiments were conducted over a 48-hour period, which is much shorter than the timescale over which CAVD typically develops. However, the acute response observed and described in this study is relevant as it may shape the long-term processes involved in the formation of calcific lesions. In addition, the experiments were performed with porcine valve leaflets due to well-characterized antibody specificities and the extensive use of this tissue source in previous valvular mechanobiology studies. However, given the intricate differences between porcine and human valve tissues, the translation of the present findings to human valves should be done with caution.

**Figure 8:**
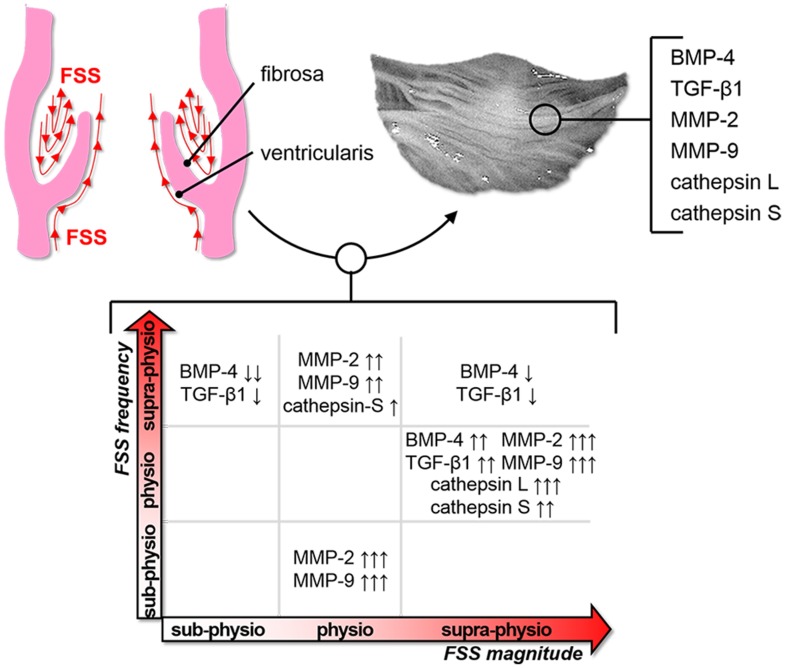
Results summary. Upregulation and downregulation of early markers of CAVD in response to FSS abnormalities (↓↓: 50%–75% expression vs. fresh tissue; ↓: 75%–100% expression; ↑: 100%–200% expression; ↑↑: 200%–500% expression; ↑↑↑: >500% expression; p<0.05 for all results).

In conclusion, this study demonstrates the sensitivity of valve leaflets to FSS abnormalities and the ability of supra-physiologic FSS magnitude or sub-/supra-physiologic FSS frequency to upregulate key CAVD mechanisms such as paracrine signaling and ECM degradation (Fig. 8). The results provide new insights into the contribution of hemodynamic stress alterations to valvular disease and their potential use as predictors of CAVD progression. Further investigation is required to elucidate the mechanisms of FSS transduction and their potential synergies with other valvular pathogenic factors.
